# New Books

**Published:** 2005-12

**Advances in Air Pollution Modeling for Environmental Security: Proceedings of the NATO Advanced Research Workshop**

István Faragó, Krassimir Georgiev, Ágnes Havasi, eds.

New York:Springer-Verlag, 2005. 406 pp. ISBN: 1-4020-3350-8, $84.95

**Advances in Arsenic Research: Integration of Experimental and Observational Studies and Implications for Mitigation**

Peggy A. O’Day, Dimitrios Vlassopoulos, Xiaoguang Meng, Liane G. Benning, eds.

Cary, NC:Oxford University Press, 2005. 450 pp. ISBN: 0-8412-3913-4, $159.50

**Applied Research in Environmental Economics**

Christoph Böhringer, Andreas Lange, eds.

New York:Springer-Verlag, 2005. 314 pp. ISBN: 3-7908-1587-X, $84.95

**Biomeasurement: Understanding, Analysing, and Communicating Data in the Biosciences**

Dawn Hawkins

Cary, NC:Oxford University Press, 2005. 312 pp. ISBN: 0-19-926515-1, $35

**Comparative Genomics**

Aoife McLysaght, Daniel H. Huson, eds.

New York:Springer-Verlag, 2005. 167 pp. ISBN: 3-540-28932-1, $58

**Dimensions in Environmental and Ecological Economics**

Nirmal Chandra Sahu, Amita Kumari Choudhury

Andhra Pradesh, India:Universities Press, 2005. 612 pp. ISBN: 81-7371-463-0, $16.17

**DNA Conformation and Transcription**

Takashi Ohyama, ed.

New York:Springer-Verlag, 2005. 220 pp. ISBN: 0-387-25579-6, $134

**Ethical Dimensions of Health Policy**

Marion Danis, Carolyn Clancy, Larry R. Churchill, eds.

Cary, NC:Oxford University Press, 2005. 424 pp. ISBN: 0-19-530083-1, $34.50

**Global Warming—Myth or Reality?**

Marcel Leroux

New York:Springer-Verlag, 2005. 510 pp. ISBN: 3-540-23909-X, $129

**Managing Natural Wealth: Environment and Development in Malaysia**

Jeffrey R. Vincent, Rozali Mohamed Ali

Washington, DC:RFF Press, 2005. 452 pp. ISBN: 1-933115-20-3, $85

**Nuclear Hazards in the World: Field Studies on Affected Populations and Environments**

Jun Takada

New York:Springer-Verlag, 2005. 134 pp. ISBN: 3-540-25272-X, $89.95

**Plows, Plagues, and Petroleum: How Humans Took Control of Climate**

William F. Ruddiman

Princeton, NJ:Princeton University Press, 2005. 272 pp. ISBN: 0-691-12164-8, $24.95

**Presenting and Representing Environments**

Graham Humphrys, Michael Williams, eds.

New York:Springer-Verlag, 2005. 218 pp. ISBN: 1-4020-3813-5, $129

**Progress in Preventing Childhood Obesity: Focus on Schools—Brief Summary: Institute of Medicine Regional Symposium**

Committee on Progress in Preventing Childhood Obesity

Washington, DC:National Academies Press, 2005. 24 pp. ISBN: 0-309-10040-2, $10.80

**Questions and Answers in Environment Science**

S.K. Basu, A.K. De

Andhra Pradesh, India:Universities Press, 2005. 396 pp. ISBN: 81-7371-547-5, $4.59

**Strategic Planning in Environmental Regulation: A Policy Approach That Works**

Steven Cohen, Sheldon Kamieniecki, Matthew A. Cahn

Cambridge, MA:MIT Press, 2005. 304 pp. ISBN: 0-262-03341-0, $60

**The Coupling of Climate and Economic Dynamics: Essays on Integrated Assessment**

Alain Haurie, Laurent Viguier, eds.

New York:Springer-Verlag, 2005. 381 pp. ISBN: 1-4020-3424-5, $169

**The Laboratory Rat, 2nd ed.**

Mark A. Suckow, Steven Weisbroth, Craig L. Franklin, eds.

Burlington, MA:Elsevier, 2005. 928 pp. ISBN: 0-12-074903-3, $139.95

**The Mediterranean Sea**

Alain Saliot, ed.

New York: Springer-Verlag, 2005. 414 pp. ISBN: 3-540-25018-2, $249

**Toxicological Evaluation of Certain Veterinary Drug Residues in Food**

Joint FAO/WHO Expert Committee on Food Additives

Geneva:World Health Organization Press, 2005. 189 pp. ISBN: 92-4-166053-8, $36

